# Systemic Capillary Leak Syndrome associated with hypovolemic shock and compartment syndrome. Use of transpulmonary thermodilution technique for volume management

**DOI:** 10.1186/1757-7241-18-38

**Published:** 2010-07-05

**Authors:** Bernd Saugel, Andreas Umgelter, Friedrich Martin, Veit Phillip, Roland M Schmid, Wolfgang Huber

**Affiliations:** 1 II. Medizinische Klinik und Poliklinik, Klinikum rechts der Isar der Technischen Universität München, Ismaninger Str. 22, D-81675 München, Germany; 2 Klinik München Perlach, Schmidbauer Str. 44, D-81737 München, Germany

## Abstract

Systemic Capillary Leak Syndrome (SCLS) is a rare disorder characterized by increased capillary hyperpermeability leading to hypovolemic shock due to a markedly increased shift of fluid and protein from the intravascular to the interstitial space. Hemoconcentration, hypoalbuminemia and a monoclonal gammopathy are characteristic laboratory findings. Here we present a patient who suffered from SCLS with hypovolemic shock and compartment syndrome of both lower legs and thighs. Volume and catecholamine management was guided using transpulmonary thermodilution. Extended hemodynamic monitoring for volume and catecholamine management as well as monitoring of muscle compartment pressure is of crucial importance in SCLS patients.

## Backround

Systemic Capillary Leak Syndrome (SCLS) is a rare disorder characterized by unexplained, often recurrent, non sepsis-related episodes of increased capillary hyperpermeability leading to hypovolemic shock due to a markedly increased shift of fluid and protein from the intravascular to the interstitial space. Hemoconcentration, hypoalbuminemia and a monoclonal gammopathy (IgG class monoclonal gammopathy predominates, with either kappa or lambda light chains) are the characteristic laboratory findings. SCLS was first described in 1960 by Clarkson et al. [1]. Common clinical manifestations of SCLS are diffuse swelling, weight gain, renal shut-down and hypovolemic shock. Here we present a patient who suffered from SCLS with hypovolemic shock and compartment syndrome of both lower legs and thighs. In this patient volume and catecholamine management was guided using transpulmonary thermodilution.

## Case Presentation

**Figure 1:**
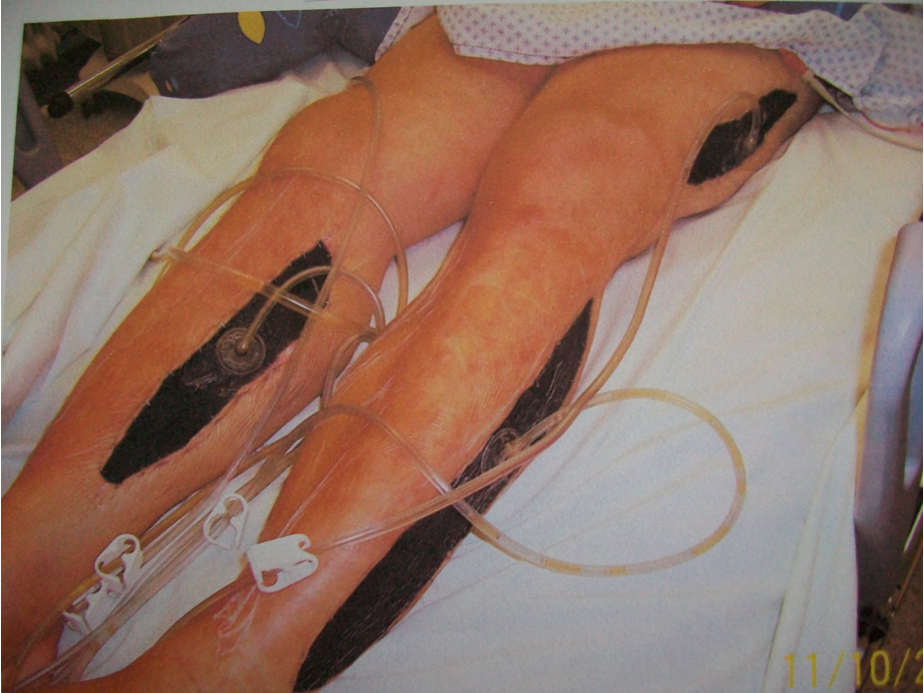
**Compartment syndrome of both lower legs and both thighs secondary to Systemic Capillary Leak Syndrome (SCLS)**. Decompressive fasciotomy

A 41-year-old male with compartment syndrome of both lower legs and thighs was transferred to our intensive care unit (ICU) (hospital B) after emergency decompressive fasciotomy in another hospital (hospital A) the previous day (fig. 1).

On admission to hospital A the previous day the patient had presented with severe muscle pain in the legs and a 2-week history of flu-like illness and sore throat with fever up to 39°C, which had been treated with moxifloxacin for several days. On initial physical examination signs of massive dehydration were present (heart rate 102/min; blood pressure 65/50 mmHg, temperature 37.1°C).

Extensive fluid resuscitation was initiated (15 L on hospital day 1). Previous medical history was unremarkable. The patient was working as a policeman and had been to Italy three weeks prior to admission. He reported playing in a football tournament one week previously.

Blood biochemistry indicated severe hemoconcentration (hemoglobin 22.3 g/dL, hematocrit 60.4%), hypoproteinemia (serum total protein 2.3 g/dL) and acute kidney failure (creatine 1.6 mg/dL, blood urea nitrogen 37 mg/dL). Markers of inflammation were only slightly altered (white blood cell count 15,900/μL, C-reactive protein 1.2 mg/dL, procalcitonin < 0.5 μg/L) and not suggestive of sepsis. Platelet count was normal. Differential blood count indicated no sign of hematologic disorders. Electrolytes were normal (sodium 133 mmol/L, potassium 4.6 mmol/L). Parameters of cholestasis and aminotransferases were not altered (bilirubin 0.7 mg/dL, alkaline phosphatase 66 U/L, gamma-glutamyl transferase 60 U/L, aspartate aminotransferase 32 U/L and alanine aminotransferase 39 U/L). Arterial blood-gas analysis showed the following: pH 7.06, pCO2 43 mmHg, pO2 91 mmHg, bicarbonate 11.9 mmol/L, anion gap 11.6 mmo/L. Creatine kinase was normal (124 U/L) on hospital day 1 and rose to over 7000 U/L on day 2 (day of admission to our ICU).

Chest radiography indicated a small right-sided pleural effusion. Echocardiography and abdominal ultrasound did not reveal any pathological findings. Lower extremity duplex sonography was performed showing no signs of venous thrombosis. The electrocardiogram was normal.

Although blood chemistry did not indicate an inflammatory constellation, an initial diagnosis of suspected sepsis with unknown focus was made (differential diagnosis: necrotizing fasciitis). Antibiotics (meropenem, clindamycin, penicillin) were administered. Measurement of pretibial compartment pressure and thigh compartment pressure by direct manometry revealed 100 mmHg and 44 mmHg, respectively. Decompressive fasciotomy of both lower legs and both thighs was performed and the patient was transferred to our ICU (hospital B) on hospital day 2 for further treatment.

On arrival to our ICU the patient was sedated, the trachea was intubated (since the fasciotomy) and the lungs were mechanically ventilated (controlled ventilation, respiratory rate on ventilator 20/min, PEEP 8 cmH2O, mean airway pressure 13 cmH2O, FiO2 0.65). Signs of protracted hypovolemic shock (arterial pressure 95/50 mmHg, heart rate 120 bpm, norepinephrine administration 0.13 μg/kg/min) were present. Laboratory tests on admission to our ICU showed the following: hemoglobin 12.9 g/dL, hematocrit 37.4%, white blood cell count 19,620/μL, platelet count 174,000/μL, creatine 1.5 mg/dL, blood urea nitrogen 21 mg/dL, C-reactive protein 2.1 mg/dL, procalcitonin 0.8 μg/L, sodium 138 mmol/L, potassium 5.2 mmol/L, bilirubin 0.2 mg/dL, alkaline phosphatase 20 U/L, gamma-glutamyl transferase 18 U/L, aspartate aminotransferase 147 U/L and alanine aminotransferase 54 U/L), lactate 4.6 mmol/L, blood gas analysis: pH 7.37, pCO2 32 mmHg, pO2 77 mmHg, bicarbonate 19.1 mmol/L, anion gap 5.6 mmo/L. Creatine kinase was 7,624 U/L (maximum value on hospital day 4: 29,195 U/L).

**Figure 2:**
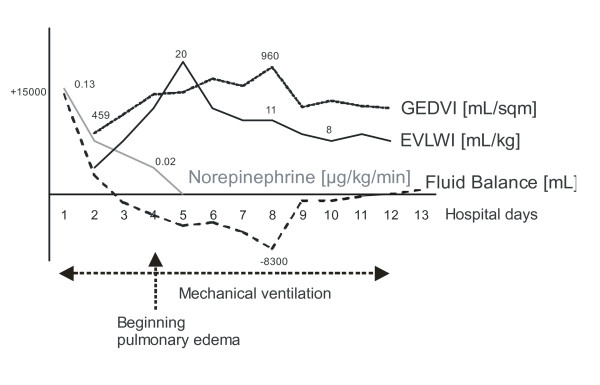
**Time course of fluid balance, extra-vascular lung water index (EVLWI), global end-diastolic volume index (GEDVI), and norepinephrine administration**.

Invasive hemodynamic monitoring using the transpulmonary thermodilution technique (PiCCO-2-device, Pulsion Medical Systems AG, Munich, Germany) was initiated. The preload parameter, global end-diastolic volume index (GEDVI) was then 459 mL/sqm (n: 680-800 mL/sqm) despite previous aggressive fluid resuscitation. Moreover, stroke volume variation (SVV; a dynamic parameter that can be assessed in patients with sinus rhythm and controlled ventilation) indicated intravascular hypovolemia and volume responsiveness (SVV 19%; n: < 10%). Further extensive fluid resuscitation and norepinephrine administration was initiated (fig. 2). On the following days, the patient continued to require catecholamine therapy to maintain a mean arterial pressure above 65 mmHg. Although the patient produced only 300 mL of urine on the first day at our ICU, hemodialysis was not required as urinary flow rate increased markedly and creatine and blood urea nitrogen values declined (maximum values: creatine 1.7 mg/dL, blood urea nitrogen 37 mg/dL) after fluid resuscitation.

Extensive tests for possible causes of hypovolemic shock and compartment syndrome were initiated. Cultures from blood, urine, pleural fluid, wound smear and central venous and arterial line catheters were tested for bacteria, fungi and mycobacterium, but were found to be sterile. Serological tests for HIV 1&2 and Leptospira as well as Influenza A/B-RNA testing by PCR were negative. Tests for antinuclear antibodies and antibodies to DNA did not reveal pathological results. Histopathology, enzyme histochemistry and electron microscopy after muscle biopsy showed normal muscle fibers without signs of muscle necrosis, myolysis, myositis or fasciitis. On electromyography no pathologic spontaneous activity was seen. The mitochondrial respiratory chain enzymes (complexes I-IV) showed normal activity. Serum IgG, IgA and IgM values were normal (727 mg/dL, 108 mg/dL and 57 mg/dL, respectively).

The antibiotic therapy started in hospital A (meropenem, clindamycin, penicillin) was continued for five more days. Then the patient was treated with piperacillin/tazobactam for another 6 days. The patient was treated with hydrocortisone (288 mg/day) for suspected septic shock for the first 6 days.

Over the following days the hypovolemic shock and edema gradually subsided under volume management (volume resuscitation with crystalloid fluid) based on transpulmonary thermodilution data and norepinephrine administration (fig. 2). In three surgical procedures the fascias of both lower legs and thighs were completely closed.

Regarding hemodynamic stabilisation, in parallel to improving GEDVI and SVV through volume loading, the extra-vascular lung water index (EVLWI) also increased (20 mL/kg; n = 3-7 mL/kg), decreasing the pO2/FiO2-ratio. There were also clinical and radiological signs of pulmonary edema developing on hospital day 4. Therefore a more restrictive volume balance including the application of diuretics was initiated resulting in markedly improved gas exchange. The tracheal tube was removed on hospital day 11 and the patient was transferred to a normal ward on hospital day 14. Serum protein immunoelectrophoresis then indicated paraprotein of the IgG kappa type. A diagnosis of idiopathic SCLS (Clarkson's disease) was made retrospectively. Two weeks after transfer to the normal ward the patient was discharged to rehabilitation.

## Conclusion

SCLS is a very rare disorder with a high mortality rate. It is characterized by increased capillary permeability resulting in hypovolemic shock due to a marked shift of fluid and protein from the intravascular to the extravascular space. Laboratory findings include hemoconcentration, hypoproteinemia and a monoclonal gammopathy [2]. SCLS was first described in 1960 by Clarkson et al. [1]. The median age for the first SCLS-manifestation is 46 years with no sex-related difference [3]. Hard physical work several days before SCLS-symptoms and flu-like-illness at the beginning of a SCLS-episode has been described in several case reports [3,4]. However the pathogenesis of SCLS is still unknown. Involvement of interleukin-2, classic pathway complement or stimulation of 5-lipooxygenase-pathway have been suggested [5-7]. The relationship between monoclonal protein and SCLS has also not been clarified. Plasma shift into the extravascular space and muscle can result in a markedly increased muscle compartment pressure and pressure induced muscle damage [8-10]. Documentation of increased muscular tension and compartment pressure can be performed by manometry. Since the risk of ischemic muscle necrosis increases markedly as compartment pressure increases above the mean arterial pressure, fasciotomy should be performed in cases of SCLS with hypotension and severe compartment syndrome. Pulmonary edema, probably induced by intravascular overloading in combination with recruitment of the initially extravasated fluids, has been described in patients with SCLS [3]. In our case report signs of pulmonary edema were present on hospital day 4 illustrating the importance of switching from the management of acute hypovolemia to management of severe fluid overload using modern hemodynamic monitoring tools.

In general optimization of intravascular volume status under consideration of pulmonary hydration is of central importance in the treatment of critically ill patients. Clinical parameters such as filling of the jugular veins (intravascular space), presence of leg edema (interstitium), ascites or pleural effusions ("third space") are still the first cornerstones in the estimation of hemodynamics and pulmonary hydration. However, according to the few studies investigating this issue, the utility of most clinical signs for the estimation of volume status might be limited due to poor specificity and sensitivity, when compared to invasive procedures [11,12]. In most ICU patients CVP can be determined easily and soon after admission. However, there is data demonstrating a poor capacity of CVP to predict the hemodynamic response to a fluid challenge [13]. Regarding more invasive techniques, transpulmonary thermodilution and pulse contour analysis are established for assessment of cardiac index, preload, volume responsiveness and pulmonary hydration [14-16]: Besides cardiac index, these techniques provide *volumetric *parameters such as GEDVI as well as *dynamic *variables of preload such as SVV for the assessment of volume responsiveness. The use of *dynamic *variables of preload is restricted to patients with sinus rhythm *and *controlled ventilation. By contrast, transpulmonary thermodilution-derived *volumetric *parameters can be used regardless of sinus rhythm and controlled ventilation to predict fluid responsiveness. Moreover, transpulmonary thermodilution accurately allows measurement of EVLWI to quantify the degree of pulmonary edema in critically ill patients.

The comparison between transpulmonary thermodilution and pulmonary artery catheter technology is still a matter of debate.

Transpulmonary thermodilution is less invasive than pulmonary thermodilution using a Swan-Ganz-catheter because it does not require the insertion of a catheter in the pulmonary artery but only a central venous and an arterial catheter (that is also needed in patients monitored with pulmonary thermodilution).

The pulmonary artery catheter is still considered to be the gold standard for assessment of cardiac index and systemic vascular resistance index. However, there is increasing data that pulmonary artery wedge pressure is not appropriate for assessment of preload and prediction of volume responsiveness, particularly in ICU patients with invasive mechanical ventilation and/or increased intra-abdominal pressure [17].

In numerous studies transpulmonary thermodilution-derived dynamic and volumetric variables of preload have been demonstrated as superior indicators of volume responsiveness as compared to pressures such as pulmonary artery wedge pressure and central venous pressure [14,18,19].

Regarding the presented case, in addition to catecholamine administration, transpulmonary thermodilution-guided volume-management regarding decreased GEDVI as valuable marker of volume deficiency and increased EVLWI as "upper threshold" for further volume resuscitation proved as very useful tool in this patient who's hydration status was difficult to judge using clinical criteria.

Several studies have also suggested that corticosteroid may be useful when the capillary leak is initiated by cytokine-mediated endothelial damage [3,20]. Treatment with terbutalin, theophylline and immunglobulines has been shown to be effective for decreasing the incidence and severity of SCLS episodes [2,21,22]. Terbutalin and theophyllin diminish the increment of bradikinin-mediated capillary permeability by an increase of cyclic adenosine monophosphate [9]. There are two reports regarding patients who developed multiple myeloma after the diagnosis of SCLS [23]. In patients with monoclonal gammopathy of undetermined significance, the risk of progression to multiple myeloma at 25 year follow-up is around 30% [24]. Therefore, annual surveillance for multiple myeloma in patients with SCLS should be recommended.

In conclusion the reported case shows the importance of extended hemodynamic monitoring for volume and catecholamine management as well as the importance of monitoring muscle compartment pressure in SCLS patients.

## Consent

Written informed consent was obtained from the patient for publication of this case report and any accompanying images. A copy of the written consent is available for review by the Editor-in-Chief of this journal.

## List of abbreviations

EVLWI: extra-vascular lung water index; GEDVI: global end-diastolic volume index; ICU: intensive care unit; SCLS: Systemic Capillary Leak Syndrome; SVV: stroke volume variation.

## Competing interests

The authors declare that they have no competing interests.

## Authors' contributions

BS, AU, FM and VP contributed to the conception and design of the case description. They were responsible for acquisition, analysis and interpretation of data regarding this case report. BS drafted the manuscript. RMS and WH participated in its design and coordination and helped to draft the manuscript. All authors read and approved the final manuscript.
